# Chromosomal miscarriage and pregnancy outcomes in recurrent pregnancy loss

**DOI:** 10.1530/RAF-25-0052

**Published:** 2025-12-08

**Authors:** Yuxin Yang, Xiaoran Zhang, Yingying Zhang, Miaoxian Ou, Can Wang, Youhui Lu, Lu Luo, Qiong Wang

**Affiliations:** ^1^ Department of Obstetrics and Gynecology, Reproductive Medical Center, First Affiliated Hospital of Sun Yat-Sen University, Guangzhou, China; ^2^ Department of Obstetrics and Gynecology, Guizhou Hospital of The First Affiliated Hospital of Sun Yat-Sen University, Obstetrics and Gynecology, Guizhou, China; ^3^ Guangdong Provincial Key Laboratory of Reproductive Medicine, Guangzhou, China

**Keywords:** recurrent pregnancy loss, pregnancy outcome, embryonic chromosomal abnormality, predictive model

## Abstract

**Abstract:**

This prospective cohort study aimed to assess whether a history of embryonic chromosomal abnormality affects subsequent pregnancy outcomes in women with unexplained recurrent pregnancy loss. A total of 233 women with unexplained recurrent pregnancy loss who conceived naturally were included. Participants were categorized according to the chromosomal status of prior miscarriages. A multivariate logistic regression model was constructed to predict live birth based on these categories and observed pregnancy outcomes. Among the 233 included women, 171 (73.4%) had a live birth, while 62 (26.6%) experienced early pregnancy loss. Among women with one abnormal chromosomal miscarriage (*n* = 110), 94 (85.5%) had a live birth, accounting for 40.3% of the total cohort. In contrast, in the one normal chromosomal miscarriage group (*n* = 77), 47 (61.0%) achieved live birth, representing 20.2% of the total. The model identified a history of one chromosomally abnormal miscarriage (odds ratio = 3.88, 95% confidence interval: 1.87–8.06) and history of two chromosomally abnormal miscarriages (odds ratio = 8.93, 95% confidence interval: 1.07–74.55) as significant predictors of live birth. The predictive model achieved an area under the curve of 0.73 in the training dataset and 0.75 in the testing dataset. A history of embryonic chromosomal miscarriage may reflect improved pregnancy prospects and inform clinical counseling in unexplained recurrent pregnancy loss.

**Lay summary:**

Women with unexplained recurrent miscarriage often wonder whether their history will affect future natural pregnancies. Chromosomal abnormalities in embryos are a leading cause of miscarriage, but the impact of such a history on later outcomes has remained unclear. We studied 233 women with repeated unexplained losses and assessed their subsequent pregnancy results. Overall, 73.4% achieved a live birth, while 26.6% had another early miscarriage. Interestingly, among women who had one chromosomal abnormal miscarriage, 85.5% subsequently achieved a live birth. In contrast, the live birth rate was lower in the group who had a normal chromosomal miscarriage; only 61.0% went on to achieve a live birth. These results suggest that a history of abnormal chromosomal miscarriage may indicate better chances of future pregnancy and help guide clinical care.

## Introduction

Recurrent pregnancy loss (RPL) refers to the occurrence of at least two pregnancy losses before 24 weeks of gestation, which affects approximately 5% reproductive couples (Dimitriadis *et al.* 2020). The risk of recurrence increases with each loss, significantly impacting mental and physical health (Tavoli *et al.* 2018, Ticconi *et al.* 2020). Known causes include uterine abnormalities, hormonal imbalances, autoimmune disorders, thrombophilia factors, and genetic factors, which can explain approximately 60% of all RPL patients (de Assis *et al.* 2024). Therefore, around 40% of cases in which no cause can be identified from the factors listed above are categorized as unexplained recurrent pregnancy loss (URPL). Current clinical guidelines recommend comprehensive history collection and screening for risk factors in RPL patients, followed by treatment based on screening results according to guideline recommendations (Bender Atik *et al.* 2018).

Genetic issues, particularly chromosomal abnormalities of miscarriage embryos, contribute to 50–70% of early pregnancy losses (Essers *et al.* 2023). The earlier the miscarriage occurs, the higher the rate of chromosomal abnormalities in the embryo (Romero *et al.* 2015). The majority of chromosomal abnormalities in embryos include aneuploidy, triploidy, monosomy X, and tetraploidy, while only a few are caused by chromosomal structural rearrangements and mosaicism (Soler *et al.* 2017, Melo *et al.* 2023). Even though current clinical guidelines do not routinely recommend genetic analysis of products of conception (POCs), they allow it for explanatory purposes rather than for predicting prognosis (Li *et al.* 2002, Khalife *et al.* 2019). Retrospective studies suggest that RPL patients with aneuploid miscarriages may have better pregnancy prognosis, with higher live birth rates and lower pregnancy loss rates (Carp *et al.* 2001, Yiming *et al.* 2023). However, several studies have shown that reproductive outcomes are similar between couples with chromosomally normal and abnormal miscarriages in sporadic cases and first clinical miscarriages (Murugappan *et al.* 2021, Xia *et al.* 2023). To date, prospective studies on subsequent pregnancy outcomes in URPL patients with previous embryonic chromosomal abnormal miscarriages, including the relationship between embryonic chromosomal results and live birth, are still lacking.

This prospective study sought to investigate the impact of a history of embryonic chromosomal miscarriage on subsequent pregnancy outcomes in women experiencing RPL of unexplained reasons and explore whether the history of embryonic chromosomal miscarriage could be a predictive factor for subsequent live birth.

## Materials and methods

### Study design

**Figure 1:**
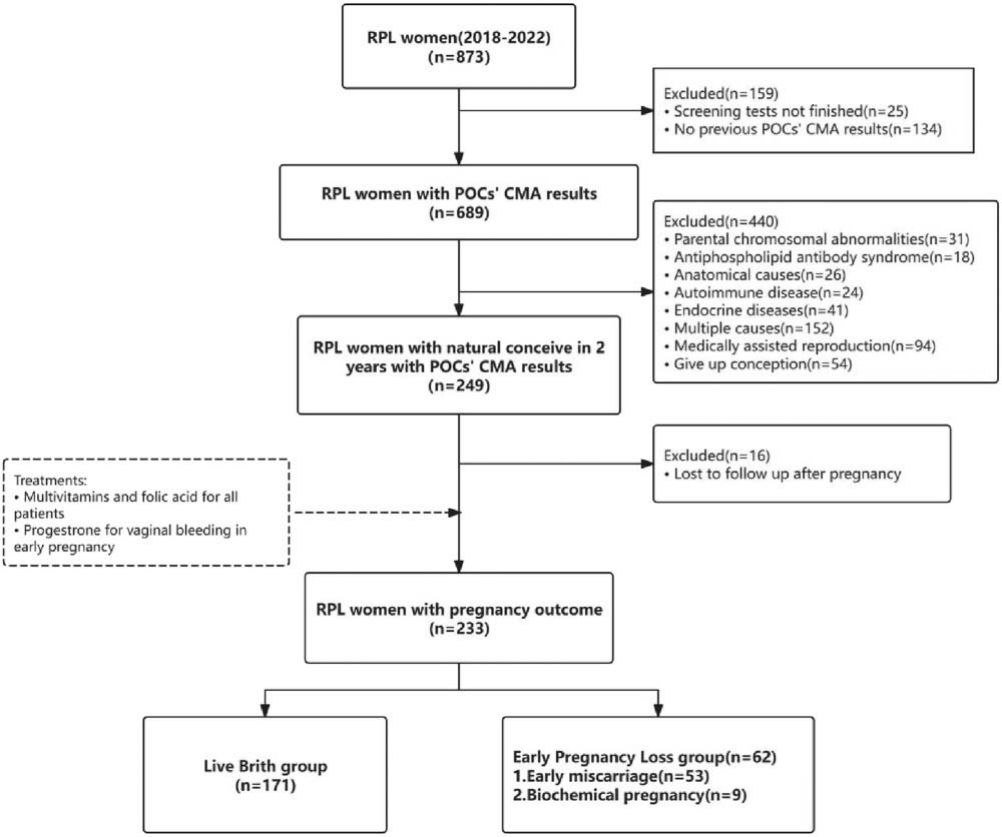
Study flow chart showing participant selection, exclusion, and pregnancy outcomes. Of 873 women with RPL assessed between 2018 and 2022, 233 with unexplained reasons who conceived naturally and completed follow-up were included. Participants were classified into a live birth group (*n* = 171) and an early pregnancy loss group (*n* = 62), including early miscarriage (*n* = 53) and biochemical pregnancy (*n* = 9). All patients received multivitamins and folic acid, and progesterone was given when early vaginal bleeding occurred.

This prospective cohort study recruited RPL patients at the Reproductive Medicine Center of the First Affiliated Hospital of Sun Yat-sen University between January 2018 and December 2022. In this study, 873 RPL women were initially assessed, and all were followed up for at least 2 years for natural pregnancy outcomes. After excluding 159 cases due to incomplete screening tests or refusal to follow-up, 689 patients with previous POC CMA results were included. Of these, an additional 440 were excluded due to a known etiology of RPL, referral for medically assisted reproduction, or giving up conception, leaving 249 RPL women who met all criteria and conceived naturally within 2 years. After excluding 16 women who were lost to follow-up, the final cohort consisted of 233 URPL participants (Fig. 1).

In this study, RPL was characterized as two or more consecutive pregnancy losses before 24 weeks of gestation. RPL women underwent the full investigations of RPL (ESHRE Guideline Group on RPL *et al.* 2023), and POC CMA testing results of previous miscarriages were collected. Investigations of RPL women were according to ESHRE RPL guidelines, including i) parental karyotyping; ii) thrombophilia screening; iii) polycystic ovary syndrome (PCOS) assessment; iv) thyroid function test; v) anti-Müllerian hormone (AMH); vi) serum prolactin level; vii) autoimmune disease screening; viii) pelvic ultrasonography; ix) endometrial biopsy for endometritis.

URPL women were included after the above screening and exclusion of known causes. To minimize heterogeneity and confounding, women who conceived through assisted reproductive techniques, including intrauterine insemination (IUI) and *in vitro* fertilization (IVF), were excluded. These procedures are associated with distinct reproductive indications and altered immunological environments due to sperm washing, which removes seminal plasma containing key immunoregulatory molecules such as TGF-β, prostaglandins, and cytokines (Cohlen *et al.* 2018, du Fossé *et al.* 2022). Limiting the analysis to spontaneous conceptions enabled a more consistent evaluation of chromosomal and immunological factors in URPL.

Exclusion criteria were: i) investigations of RPL not completed; ii) lack of CMA results of POCs; iii) known causes of RPL; iv) assisted reproductive technology for the next conception, including preimplantation genetic testing, IVF, and intracytoplasmic sperm injection (ICSI); v) intrauterine insemination (IUI); vi) giving up conception; vii) lost to follow-up.

Multivitamins and folic acid (Elevit) were given to all patients. Patients with threatened abortion were prescribed progesterone.

For the follow-up procedure, pregnancy was first confirmed by β-HCG measurement from peripheral blood after a positive urine HCG quick test; ultrasound was used to verify fetal cardiac activity during 6–8 gestational weeks, then nuchal translucency screening was performed at approximately 12 weeks. Pregnancy outcomes and pregnancy-related complications were followed up by telephone interviews or medical records every 2 months until birth. Each patient was followed up for 2 years after preparation for conception. For subsequent miscarriage cases, chromosomal analysis of the POC was proposed.

Ethical approval for this study was granted by the Ethics Committee Board at the First Affiliated Hospital of Sun Yat-sen University (2016-116). Consent was obtained from each patient or subject after full explanation of the purpose and nature of all procedures used.

### Outcome measurement

The main outcomes included live birth and early pregnancy loss. A live birth was characterized as the birth of at least one viable neonate at or beyond 24 weeks of gestation. Early miscarriage was referred to as a clinical pregnancy in which intrauterine utero demise occurred before 12 weeks. Biochemical pregnancy was considered as positive detection of human chorionic gonadotropin (hCG) in maternal blood without progression to a clinical pregnancy. Early pregnancy loss included early miscarriage and biochemical pregnancy.

The secondary outcomes were pregnancy-related complications, including threatened abortion, preterm birth, gestational hypertension or preeclampsia, gestational diabetes, spontaneous premature labor, small or large for gestational age. Threatened abortion was defined as vaginal bleeding and uterine cramping before 20 weeks of gestation (Nepyivoda & Ryvak 2020). Preterm birth was defined as the delivery of an infant occurring before the gestation period reached 37 completed weeks (Vogel *et al.* 2018). Gestational hypertension was characterized by elevated blood pressure (systolic ≥140 mmHg or diastolic ≥90 mmHg) occurring after 20 gestational weeks. Preeclampsia was subsequently characterized by the onset of proteinuria or newly developed hypertension without proteinuria, along with low platelet count, abnormal liver function, kidney dysfunction, fluid accumulation in the lungs, or neurological/visual impairments (Morikawa *et al.* 2021). Gestational diabetes was diagnosed when at least one value from a 75 g oral glucose tolerance test after a minimum of 8 h of fasting exceeded the IADPSG criteria during pregnancy (Aaron *et al.* 2018). Spontaneous premature labor was referred to as labor occurring between 20 and 37 weeks due to early contractions, membrane rupture, or cervical insufficiency (Battarbee *et al.* 2024). Postpartum hemorrhage (PPH) refers to blood loss of ≥500 mL after vaginal delivery or ≥1,000 mL after cesarean delivery, accompanied by signs of hypovolemia or hemodynamic instability (Savvidou 2023). Small for gestational age (SGA) referred to infants with birth weights below the 10th percentile for their gestational age based on standardized growth charts. Large for gestational age (LGA) described infants whose birth weights exceeded the 90th percentile for their gestational age (Aris *et al.* 2019).

### Statistical analysis

Data were processed and analyzed utilizing SPSS version 26 and R version 4.4.0. The Shapiro–Wilk normality test was conducted to assess the normality of continuous variables. For continuous variables exhibiting a normal distribution, the results are reported as mean ± standard deviation and assessed with an independent samples *t*-test. Conversely, for variables exhibiting a non-normal distribution, data are shown as median (1st quartile, 3rd quartile) and were assessed using the Mann–Whitney U test. Counts and percentages were expressed for categorical variables, and the chi-square or Fisher’s exact tests were conducted. A total of 22 candidate variables were involved in this study: maternal age, body mass index (BMI), male age, number of parity, number of previous miscarriage, number of biochemical pregnancy, one abnormal chromosomal miscarriage, one normal chromosomal miscarriage, one normal and one abnormal chromosomal miscarriage, two abnormal chromosomal miscarriages, two normal chromosomal miscarriages, number of induced abortions, months from the last embryonic CMA results to this pregnancy, mean gestational week of previous miscarriage, basal follicle-stimulating hormone (bFSH), AMH, basal luteinizing hormone (bLH), basal prolactin (bPRL), thyroid peroxidase antibody (TPOAb), antinuclear antibody (ANA) level, anti-thyroglobulin antibody (TGAb), fasting blood glucose (GLU) level, and thyroid-stimulating hormone (TSH) level. Binary variables were analyzed using the phi coefficient, continuous variables were assessed using the Pearson correlation coefficient, and those with correlations over 0.7 were excluded to reduce collinearity. Univariate regression analysis and multivariate logistic regression analysis with stepwise method (backwards) were conducted for the included variables. Forest plots based on multivariate logistic regression regarding variables associated with subsequent live birth were plotted. The x-axis was plotted on a logarithmic scale to appropriately represent the wide range of OR values.

A predictive model was developed based on multivariate logistic regression, and a nomogram was conducted to explore the relationship between previous abnormal/normal embryonic miscarriage, and subsequent live birth. The participants were randomly divided, with 70% allocated to the training dataset for model construction and the remaining 30% assigned to the testing dataset to evaluate the final model’s performance. Model performance was evaluated in terms of discrimination, using the area under the curve, and the model’s calibration was evaluated by comparing predicted probabilities with observed outcomes using calibration plots. To determine the best trade-off between sensitivity and specificity, we calculated the Youden index. In addition, the model’s explanatory capacity was evaluated using the Nagelkerke pseudo-*R*^2^, while its goodness-of-fit was analyzed with the Hosmer–Lemeshow test.

## Results

### Patients’ information and baseline characteristics

A total of 279 POC CMA results were collected from 233 URPL patients, of which 163 had abnormal results (details shown in Supplementary Table S1 (see section on Supplementary materials given at the end of the article)) and 116 had normal results. Overall, 171 (73.4%) URPL patients achieved live birth, while 62 (26.6%) experienced early pregnancy loss, including 53 (22.7%) early miscarriages and 9 (3.9%) biochemical pregnancies. Then, patients were categorized into a live birth group and an early pregnancy loss group. The flow chart is shown in Fig. 1.

The live birth group had fewer previous miscarriage compared to the early pregnancy loss group (*P* < 0.001). The percentage of URPL women with one abnormal chromosomal miscarriage was significantly higher in the live birth group than in the early pregnancy loss group (55.0 vs 25.8%), and the proportions of women with one or two normal chromosomal miscarriages were significantly lower in the live birth group than in the early pregnancy loss group (27.49 vs 48.39%, *P* = 0.003; 2.34 vs 9.68%, *P* = 0.038). No other characteristics, including maternal age, male partner age, BMI, basal female hormones, metabolic and immune-related parameters, pregnancy interval, mean gestational week, and adverse pregnancy history, showed significant differences between the two groups (Table 1).

**Table 1:** Baseline characteristics in the live birth and pregnancy loss groups. Data are presented as mean ± SD, median (Q1, Q3), or as *n* (%).

Variables	Total (*n* = 233)	Live birth group (*n* = 171)	Early pregnancy loss group (*n* = 62)	*P*
Maternal age (year)	31.07 ± 4.01	31.55 ± 3.60	30.89 ± 4.15	0.273
Male age (year)	33.08 ± 4.63	33.58 ± 4.13	32.90 ± 4.80	0.331
BMI (kg/m^2^)	20.98 ± 1.96	21.10 ± 1.80	20.94 ± 2.02	0.586
AMH (ng/mL)	3.38 (2.23, 4.67)	2.92 (2.15, 5.07)	3.46 (2.34, 4.65)	0.576
bFSH (IU/L)	5.40 (4.53, 6.17)	5.29 (4.38, 6.14)	5.43 (4.60, 6.17)	0.571
bLH (IU/L)	3.80 (2.98, 5.03)	4.05 (3.11, 4.86)	3.71 (2.92, 5.11)	0.582
bProlactin (IU/L)	19.80 (16.23, 24.36)	19.02 (16.50, 25.32)	20.11 (15.68, 23.93)	0.752
Fasting blood glucose (mmol/L)	4.80 (4.50, 5.00)	4.90 (4.60, 5.00)	4.80 (4.50, 5.10)	0.585
Fasting insulin (mU/L)	7.38 (5.45, 10.26)	7.62 (6.00, 10.20)	7.20 (5.39, 10.23)	0.760
ANA (U/mL)	3.00 (1.70, 7.62)	3.00 (1.40, 7.31)	3.00 (1.80, 8.03)	0.758
TGAB (mU/L)	0.06 (0.00, 0.40)	0.05 (0.00, 0.37)	0.07 (0.00, 0.40)	0.817
TPOAB (mU/L)	0.69 (0.33, 2.09)	1.14 (0.30, 2.69)	0.60 (0.35, 1.40)	0.251
TSH (mIU/L)	1.79 (1.30, 2.46)	1.90 (1.35, 2.48)	1.62 (1.14, 2.27)	0.353
Time from last POC CMA to pregnancy (months)	17.00 (14.00, 22.00)	16.50 (13.25, 22.00)	17.00 (14.00, 22.75)	0.389
Mean gestational week (weeks)	7.00 (6.00, 8.00)	8.00 (6.75, 8.00)	7.00 (6.00, 8.00)	0.396
No. of previous miscarriages				<0.001
2	154 (66.09)	126 (73.26)	28 (45.90)	
3	65 (27.90)	40 (23.26)	25 (40.98)	
≥4	14 (6.01)	6 (3.49)	8 (13.11)	
Parity				1.000
0	190 (81.55)	140 (81.40)	50 (81.97)	
1	40 (17.17)	30 (17.44)	10 (16.39)	
2	3 (1.29)	2 (1.16)	1 (1.64)	
No. of induced abortions				0.449
0	187 (80.26)	141 (81.98)	46 (75.41)	
1	41 (17.60)	28 (16.28)	13 (21.31)	
≥2	5 (2.15)	3 (1.74)	2 (3.28)	
No. of biochemistry pregnancy				0.899
0	195 (83.69)	145 (84.30)	50 (81.97)	
1	25 (10.73)	18 (10.47)	7 (11.48)	
≥2	13 (5.58)	9 (5.23)	4 (6.56)	
Ectopic pregnancy history				
No	227 (97.42)	167 (97.66)	60 (96.77)	1.000
Yes	6 (2.58)	4 (2.34)	2 (3.23)	
Chromosomal miscarriages				
Once abnormal				<0.001
No	123 (52.79)	77 (45.03)	46 (74.19)	
Yes	110 (47.21)	94 (54.97)	16 (25.81)	
Once normal				0.003
No	156 (66.95)	124 (72.51)	32 (51.61)	
Yes	77 (33.05)	47 (27.49)	30 (48.39)	
Once normal and once abnormal				0.111
No	214 (91.85)	160 (93.57)	54 (87.10)	
Yes	19 (8.15)	11 (6.43)	8 (12.90)	
Twice abnormal				0.249
No	216 (92.70)	156 (91.23)	60 (96.77)	
Yes	17 (7.30)	15 (8.77)	2 (3.23)	
Twice normal				0.038
No	223 (95.71)	167 (97.66)	56 (90.32)	
Yes	10 (4.29)	4 (2.34)	6 (9.68)	

### History of abnormal chromosomal miscarriage could be a predictor of subsequent live birth

All 233 URPL patients were divided into five subgroups according to previous POC CMA results. Among the 233 patients, live births were most frequently observed in the one abnormal group (94/233, 40.3%), followed by the one normal group (47/233, 20.2%), two abnormal group (15/233, 6.4%), one abnormal and normal group (11/233, 4.7%), and two normal group (6/233, 2.6%). Early pregnancy loss occurred in 15 (6.4%), 26 (11.2%), 6 (2.6%), 2 (0.9%), and 11 (4.7%) patients from these respective groups. The one abnormal group had a significantly higher live birth rate (85.5%, *n* = 94/110) than the one normal group (61.0%, *n* = 47/77) and the one abnormal and normal group (57.9%, *n* = 11/19). Similarly, the early miscarriage rate was significantly lower in the one abnormal group (13.6%, *n* = 15/110) compared to the one normal group (33.8%, *n* = 26/77). Moreover, the biochemical pregnancy rate in the one abnormal group (6.4%, *n* = 7/110) was also lower than that in the two normal group (20.0%, *n* = 2/10). No significant differences in pregnancy-related complications were observed among the subgroups (Table 2).

**Table 2:** Pregnancy outcomes of previous recurrent pregnancy loss women with miscarriage chromosomal results. Data are presented as *n* (%).

	Once abnormal group (*n* = 110)	Once normal group (*n* = 77)	Once normal and abnormal group (*n* = 19)	Twice abnormal group (*n* = 17)	Twice normal group (*n* = 10)	*P*
Live births	94 (85.5)^a^	47 (61.0)^b^	11 (57.9)^b^	15 (88.2)^a,b^	6 (60.0)^a,b^	<0.001
Term delivery	90 (95.7)	45 (95.7)	11 (100)	15 (100)	6 (100)	
Preterm birth	4 (4.3)	2 (2.6)	0 (0)	0 (0)	0 (0)	1.000
Early miscarriage	15 (13.6)^a^	26 (33.8)^b^	7 (36.8)^a,b^	1 (5.9)^a,b^	2 (20.0)^a,b^	0.003
Biochemical pregnancy	1 (0.9)^a^	4 (5.2)^a,b^	1 (5.3)^a,b^	1 (5.9)^a,b^	2 (20.0)^b^	0.026
Threatened abortion	8 (8.5)	3 (6.4)	1 (9.1)	0 (0.0)	1 (16.7)	0.601
Pregnancy complications	28 (26)	15 (29.8)	7 (63.6)	2 (13.3)	1 (16.7)	0.096
Gestational diabetes	19 (20.2)	9 (19.1)	2 (18.2)	2 (13.3)	0 (0)	0.655
Preeclampsia or gestational hypertension	3 (3.2)	1 (2.1)	1 (9.1)	0 (0)	0 (0)	0.520
Spontaneous premature labor	3 (3.2)	3 (6.4)	2 (18.2)	0 (0)	0 (0)	0.366
Postpartum hemorrhage	1 (1.1)	2 (2.1)	0 (0)	0 (0)	0 (0)	0.733
Small/large for gestational age	2 (2.1)	1 (2.1)	2 (18.2)	0 (0)	1 (16.7)	0.087

Different letters (^a, b^) indicate significant differences between groups (*P <* 0.05).

Shared letters (^a^ or ^b^ or ^a, b^) indicate no significant difference.

**Figure 2:**
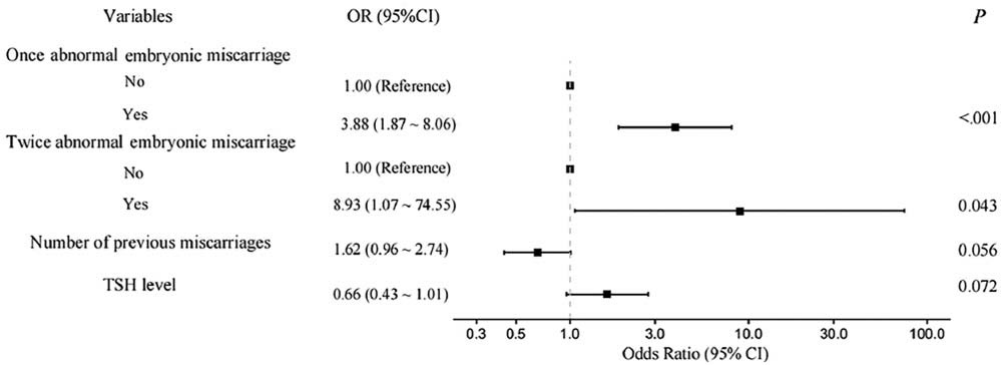
Forest plot showing results of the multivariate logistic regression analysis of variables associated with live birth in women with URPL. Odds ratios (ORs) with 95% confidence intervals (CIs) and *P* values are shown for each variable.

To explore the predictive factors for live birth in women with previous RPL, a total of 22 variables were evaluated. Initially, the history of one normal and one abnormal embryonic miscarriage was excluded from the total of 22 variables due to strong correlations with one abnormal embryonic miscarriage, one normal embryonic miscarriage, two abnormal embryonic miscarriage, and two normal embryonic miscarriages. Then, only four out of twenty-one variables were included in the final predictive model, which is presented in the forest plot (Fig. 2). Specifically, we found that a history of one abnormal embryonic miscarriage (OR = 3.88, 95% CI: 1.87–8.06, *P* < 0.001) and a history of two abnormal embryonic miscarriages (OR = 8.93, 95% CI: 1.07–74.55, *P* = 0.043) were significantly positively correlated with live birth. Conversely, the number of previous miscarriages was identified as a risk factor associated with live birth (OR = 0.66, 95% CI: 0.43–1.01), although there was no statistical significance (*P* = 0.056). TSH level was also a predictive factor for live birth without significant difference (OR = 1.62, 95% CI: 0.96–2.74, *P* = 0.072).

**Figure 3:**
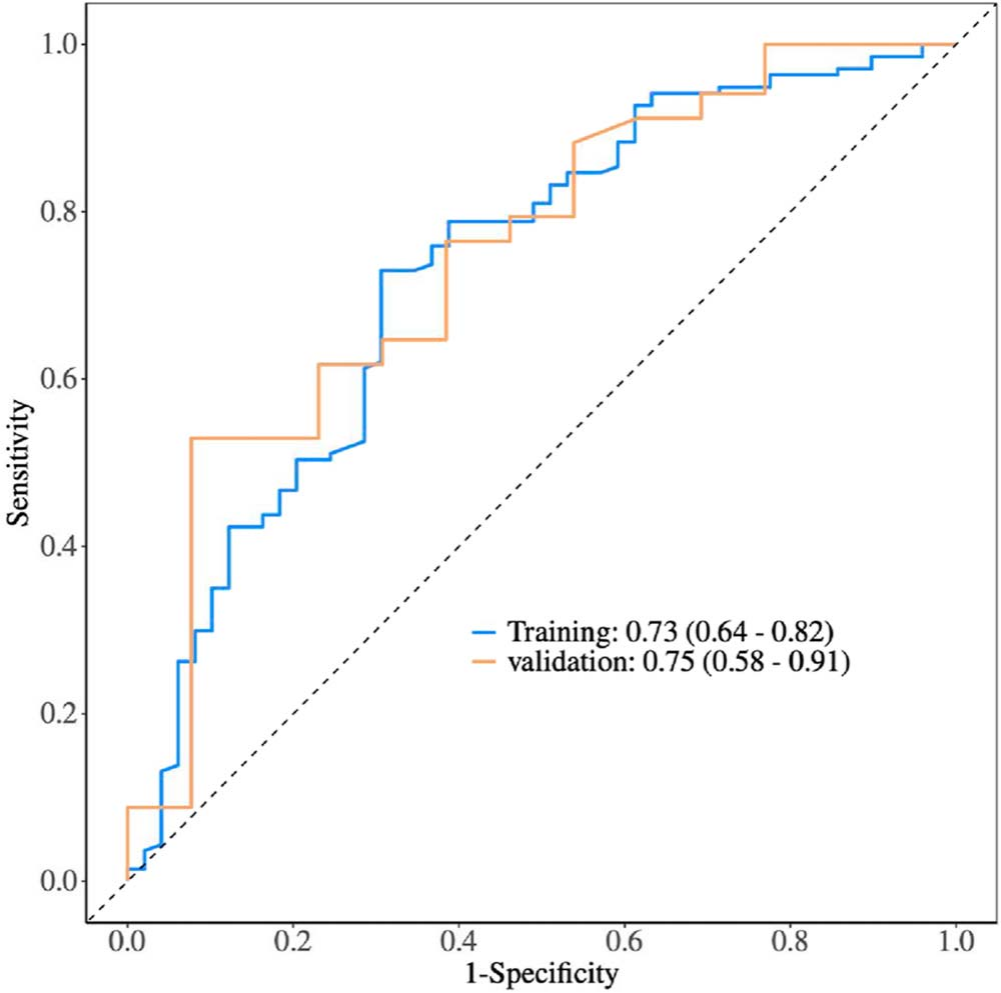
ROC curve evaluating the predictive performance of the logistic regression model for live birth. The area under the curve (AUC) was 0.73 (95% CI: 0.64–0.82) in the training dataset and 0.75 (95% CI: 0.58–0.91) in the validation dataset.

The predictive model demonstrated a receiver operating characteristic (ROC) curve area of 0.73 (95% CI: 0.64–0.82) in the training dataset and 0.75 (95% CI: 0.58–0.91) in the testing dataset, as presented in Fig. 3. No statistical difference was observed between the ROC for the training and testing datasets (*P* = 0.774). The calibration plot showed acceptable performance. For the training dataset, it achieved a Hosmer–Lemeshow *P* of 0.663, Nagelkerke pseudo-*R*^2^ of 0.238, an average discrepancy of 33.39%, and a maximum discrepancy of 95.15%, with a calibration intercept of −0.02 and a slope of 1.03 (Supplementary Fig. S1). For the testing dataset, the plot had a Hosmer-Lemeshow *P* of 0.280, Nagelkerke pseudo-*R*^2^ of 0.219, an average discrepancy of 35.01%, and a maximum discrepancy of 92.25%, with a calibration intercept of 0.06 and a slope of 0.95 (Supplementary Fig. S2). The sensitivity (SE) and specificity (SP) for the training dataset were 0.69 (95% CI: (0.56–0.82) and 0.73 (95% CI: 0.66–0.80), respectively.

**Figure 4:**
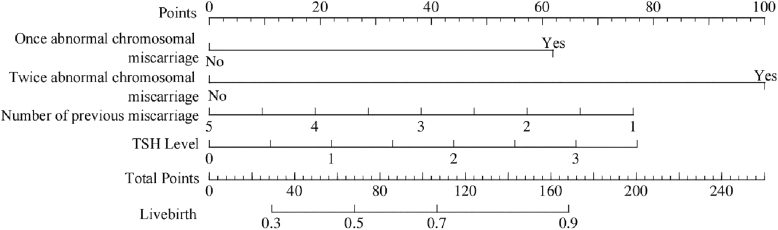
Nomogram for predicting the probability of live birth in women with URPL. Predictors included chromosomal status of previous miscarriages, number of previous miscarriages, and TSH level. Points assigned to each predictor are summed to estimate the probability of live birth.

The nomogram shows each factor’s contribution to the likelihood of live birth, with higher total scores indicating a greater probability of a subsequent live birth (Fig. 4). For both the training and testing datasets, decision curve analysis indicated that the model provided superior net benefit compared to both the treating-all and treating-none strategies over a broad range of threshold probabilities, particularly between 0.1 and 0.6, indicating its meaningful improvement in clinical decision-making (Supplementary Figs S3 and S4).

## Discussion

### Interpretations of the results

This is the first prospective study to investigate subsequent pregnancy outcomes in URPL patients with previous normal or abnormal chromosomal miscarriages. We found patients with a history of chromosomal abnormality-related miscarriages had a significantly higher subsequent live birth rate and lower miscarriage rate compared to those with chromosomally normal miscarriages. Furthermore, multivariate logistic regression showed that a history of one abnormal chromosomal miscarriage (OR = 3.88), a history of two abnormal chromosomal miscarriages (OR = 8.93), the number of previous pregnancy losses, and TSH level were important predictors for subsequent live birth in URPL women.

Few studies have examined the reproductive prognosis of RPL women with chromosomally abnormal or normal miscarriages. In sporadic miscarriages, reproductive outcomes were similar for couples with abnormal and normal chromosomal miscarriages (Xia *et al.* 2023). Likewise, the prognosis after a first clinical miscarriage was equally favorable regardless of whether the karyotype was euploid or aneuploid among infertile patients (Murugappan *et al.* 2021). However, one recent retrospective cohort study found that, after adjusting for maternal age, URPL women with aneuploid miscarriages had higher live birth rates and lower spontaneous miscarriage rates compared to those with chromosomally normal miscarriages (Yiming *et al.* 2023). Another study found that RPL patients with aneuploid miscarriages had a higher live birth rate, but the difference was not significant (Carp *et al.* 2001). Similarly, we demonstrated a significantly higher live birth rate from patients with one previous abnormal chromosomal miscarriage compared to patients with one normal or one normal plus one abnormal chromosomal miscarriage in this prospective study. Furthermore, an attention-getting observation is that the live birth rate in URPL women with one or even two normal chromosomal miscarriages was nearly 60% without additional interventions. The phenomenon that URPL patients with chromosomal abnormality-related miscarriages have better reproductive outcomes may suggest that aneuploidy arises randomly during meiosis or early embryonic development, and this might be an opportunistic event that does not impair reproductive potential in subsequent pregnancies (Siegel & Amon 2012, Sato *et al.* 2019). Importantly, these findings may provide psychological reassurance to affected patients by suggesting that a history of chromosomal abnormal miscarriage does not necessarily imply impaired reproductive potential. They may also support clinicians in offering more individualized prognostic counseling and setting realistic expectations for future pregnancy outcomes.

We found that women in the live birth group had fewer previous miscarriages compared to those in the early pregnancy loss group, and a higher number of previous miscarriages was associated with a reduced likelihood of live birth (OR = 0.66) without significant difference. This result was consistent with previous studies that showed an increasing number of miscarriages significantly reduces the likelihood of live birth (Field & Murphy 2015, Hooker *et al.* 2021). The rising proportion of chromosomally normal losses with higher miscarriage numbers highlights the potential role of maternal factors in RPL, necessitating detailed investigations beyond embryonic chromosomal analysis (Macklon *et al.* 2002, Brosens *et al.* 2022). In addition, we found that TSH level was also a predictive factor for live birth without significant difference. It is noteworthy that the patients recruited for this study had already been excluded if they had thyroid dysfunction or positive thyroid autoantibodies. Previous studies have shown that women with TSH levels outside of the normal range may face higher risks of miscarriage and adverse pregnancy outcomes compared to those with TSH levels between 0.4 and 2.49 mIU/L (Yang *et al.* 2022). However, within the normal TSH range in euthyroid individuals, the impact of preconception TSH levels on live birth outcomes remains unclear. In our study, we observed that within the normal range of TSH levels, higher TSH levels were associated with subsequent live birth. This result may be due to the small sample size and random variation, and further research is needed to clarify this relationship.

The predictive model based on multivariate logistic regression demonstrated good overall performance with an AUC for the training and testing datasets. To date, no predictive models for live birth in URPL patients with previous abnormal embryonic chromosomal miscarriages have been reported. Existing studies on predictive models for live birth in RPL with known factors, such as maternal age, number of miscarriages, antiphospholipid antibodies, and thyroid function including TSH levels (Bashiri *et al.* 2022, Dai *et al.* 2022, Li *et al.* 2024), highlight the importance of incorporating multiple dimensions to better understand live birth prognosis in RPL patients (Shulman 2012, Bashiri *et al.* 2022). In our prospective observational study, by incorporating both normal and abnormal chromosomal miscarriages, our model achieved greater comprehensiveness and clinical relevance for URPL patients. Nonetheless, the relatively small sample size in specific subgroups, particularly in patients with two chromosomally abnormal miscarriages, resulted in wide confidence intervals, which may limit the precision of the subgroup analysis. Furthermore, as the predictive model was developed and internally validated within a single-center cohort, external validation in larger, multicenter populations is necessary to confirm its predictive value and generalizability in broader clinical practice.

Chromosomal microarray analysis (CMA) has emerged as a superior technique for identifying structural genetic variations, such as microdeletions, microduplications, and unprecedented CNVs, which traditional methodologies such as karyotyping and FISH may overlook (Popescu *et al.* 2018, Sagi-Dain *et al.* 2018). All collected POC chromosomal analyses in our study were conducted using chromosomal microarray analysis (CMA), which demonstrated superior diagnostic accuracy and sensitivity (Resta & Memo 2012, Hillman *et al.* 2013). However, not all patients with normal chorionic villus results in this study underwent maternal cell contamination testing, which could lead to the misdiagnosis of abnormal embryonic chromosomes as normal (Nykel *et al.* 2021). Of the 279 POC samples from 233 women, 99 underwent maternal cell contamination (MCC) testing. Specifically, 66 of the 116 samples with normal chromosomal microarray analysis (CMA) results were tested, of which 54 showed no contamination and 12 showed low-level contamination (3–30%). Of the 163 samples with abnormal CMA results, 33 underwent MCC testing: 24 were negative, 8 had low-level contamination (3–35%), and one showed high-level contamination (85%). Although MCC testing is now standard in our center, it was not consistently performed in earlier years due to evolving laboratory protocols. While most chromosomal abnormalities detected, such as aneuploidies, are unlikely to be misinterpreted due to contamination, this limitation may still affect a small subset of cases and should be considered in interpretation. Despite this limitation, our results still indicated a higher live birth rate in abnormal chromosomal miscarriage rather than normal chromosomal miscarriage. Therefore, this design limitation may not alter our findings. Moreover, no significant difference in live birth rates was found between the aneuploidy subgroup (88/118, 74.6%) and the other abnormality subgroup (32/45, 71.1%). Due to the small sample size of non-aneuploidy patients, we did not stratify subgroups and compare pregnancy outcomes based on detailed subtypes of non-aneuploidy chromosomal abnormalities, and larger cohort studies may be needed for subtypes of detailed non-aneuploidy in the future.

## Conclusion

This prospective observational cohort study found that a history of one or two abnormal chromosomal miscarriages significantly increased the likelihood of live birth, highlighting the positive prognosis associated with abnormal chromosomal miscarriages. The predictive model demonstrated good performance with robust sensitivity and specificity, which provides a practical tool for identifying patients at higher risk of subsequent pregnancy loss and guiding clinical management. Future studies need to focus on the potential causes in URPL patients with a history of normal chromosomal miscarriage.

## Supplementary materials



## Declaration of interest

The authors declare that there is no conflict of interest that could be perceived as prejudicing the impartiality of the research reported in this study. No financial, professional, or personal relationships exist that could influence the findings presented.

## Funding

This study was supported by the Guangzhou Municipal Science and Technology Projecthttps://doi.org/10.13039/501100010256 under project number 202206010003.

## Author contribution statement

YXY and XRZ designed the methodology and wrote the original draft, MXO and YYZ interpreted and verified the data, CW and YHL programmed the statistical software, LL helped with data analysis, and QW conceptualized the study, revised, and approved the manuscript.

## Ethical approval and consent

Ethical approval for this study was granted by the Ethics Committee Board at the First Affiliated Hospital of Sun Yat-sen University (2016-116). Consent has been obtained from each patient or subject after full explanation of the purpose and nature of all procedures used.
