# Supplementary material for: Chromosomal miscarriage and pregnancy outcomes in recurrent pregnancy loss
Source: Reprod Fertil. 2025 Dec 8;6(4):e250052. doi: 10.1530/RAF-25-0052 (PMC12694013; doi:10.1530/RAF-25-0052)
Supplement: Supplementary file 1 [file supplementary_materials.pdf]

Supplementary Figure S1 Calibration plots for the training datasets

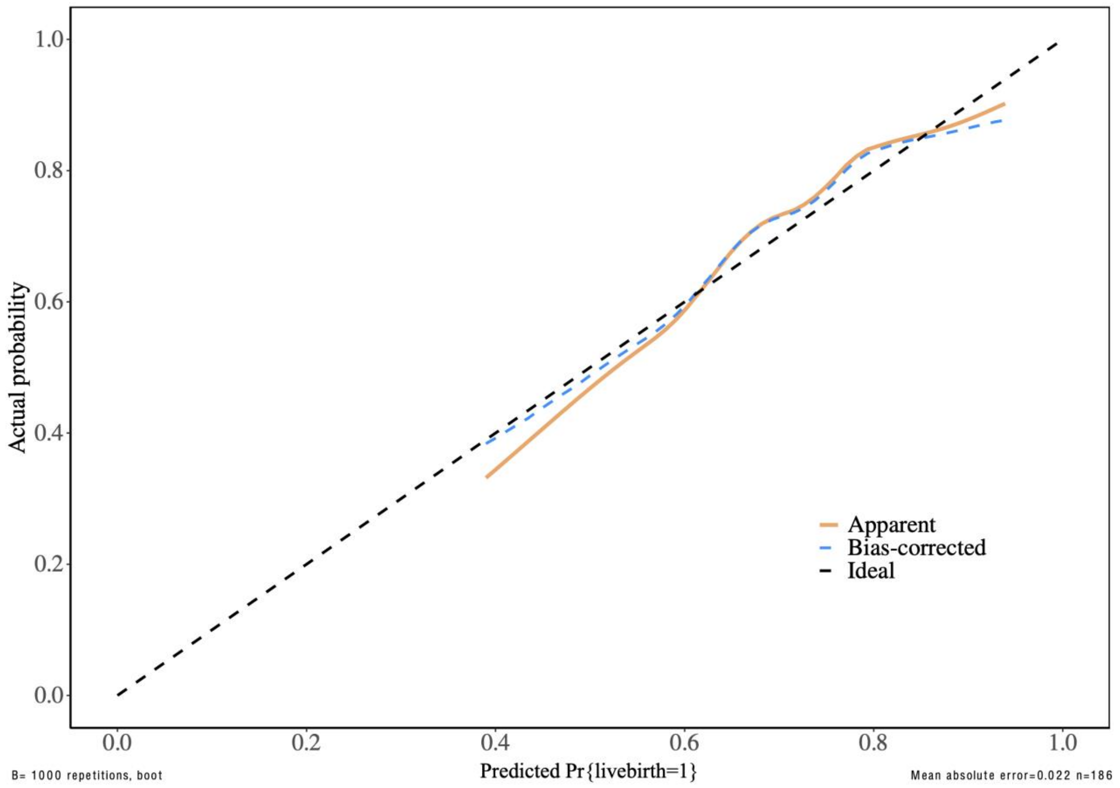

Figure legend: Calibration plot of the logistic regression model in the training dataset. The apparent curve and bias-corrected curve are shown against the ideal reference line. The model demonstrates good concordance between predicted and observed probabilities (Hosmer-Lemeshow *P* = 0.663; mean absolute error = 0.022; n = 186).

Supplementary Figure S2 Calibration plots for the test datasets

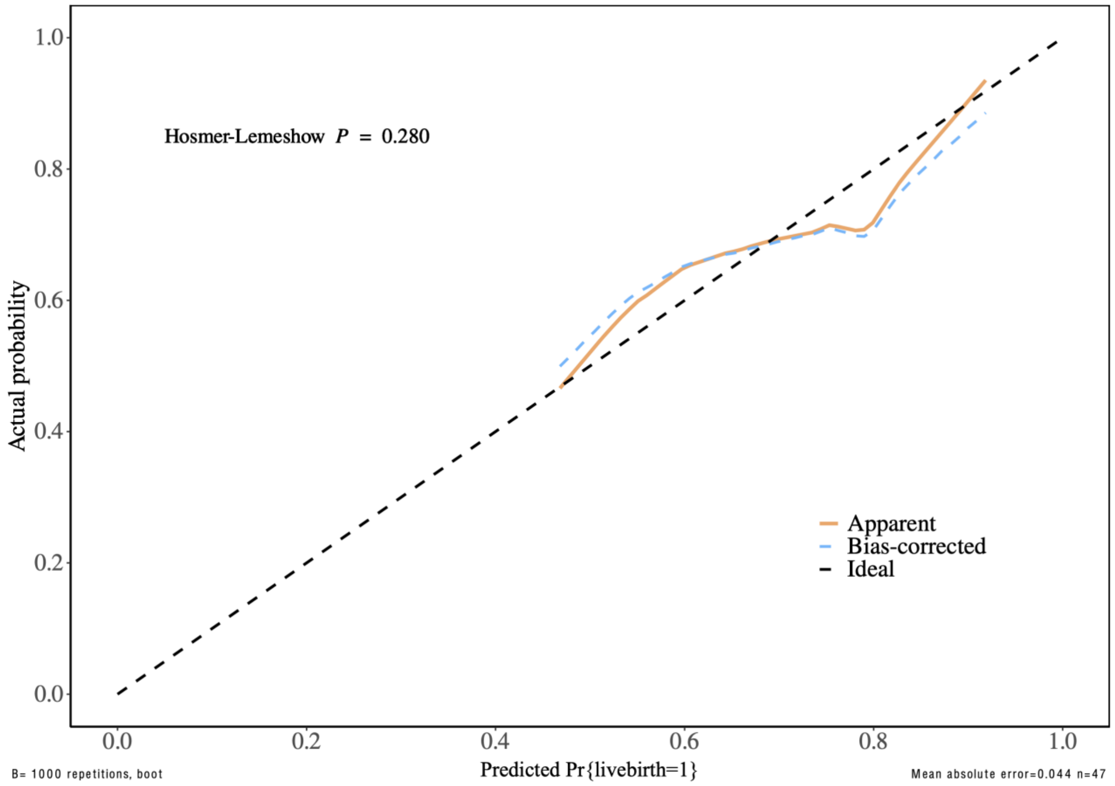

Figure legend: Calibration plot of the logistic regression model in the test dataset. The apparent curve and bias-corrected curve are shown against the ideal reference line. The model shows acceptable agreement between predicted and observed probabilities (Hosmer-Lemeshow test *P* = 0.280; mean absolute error = 0.044; n = 47).

Supplementary Figure S3 Decision Curve Analysis for the training datasets

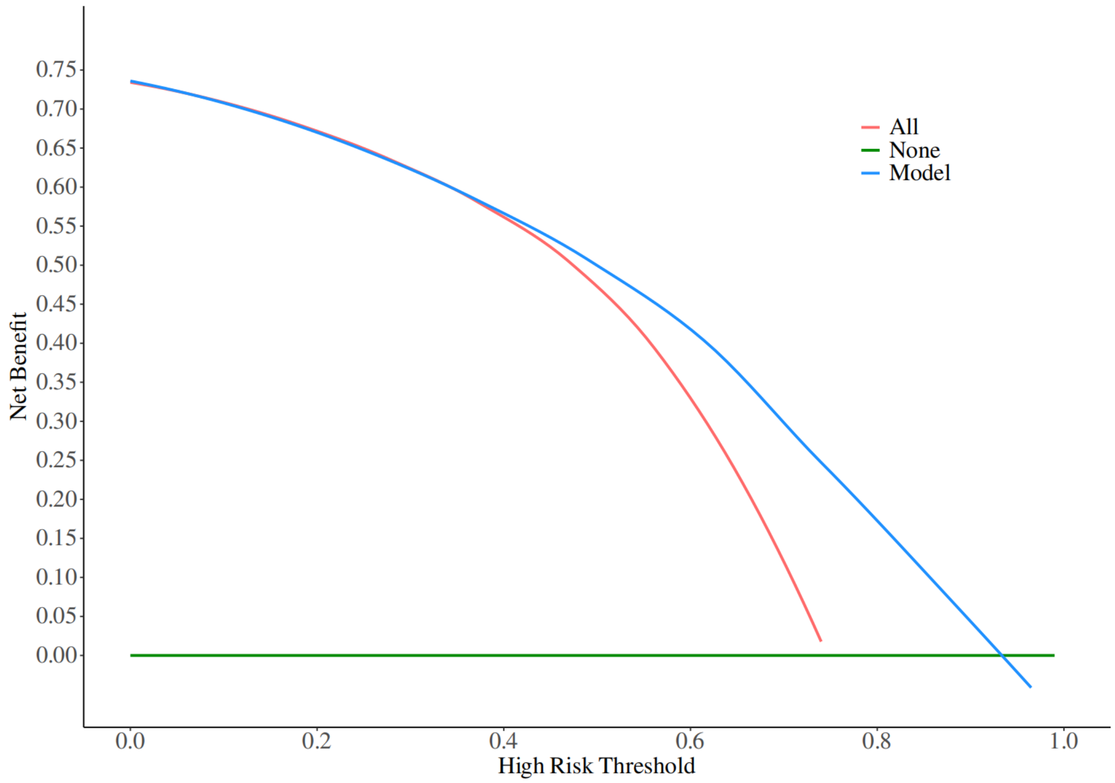

Figure legend: Decision curve analysis (DCA) for the training dataset. The blue line represents the net benefit of the prediction model across different threshold probabilities, compared with treating all patients (red line) or none (green line).

Supplementary Figure S4 Decision Curve Analysis for the test datasets

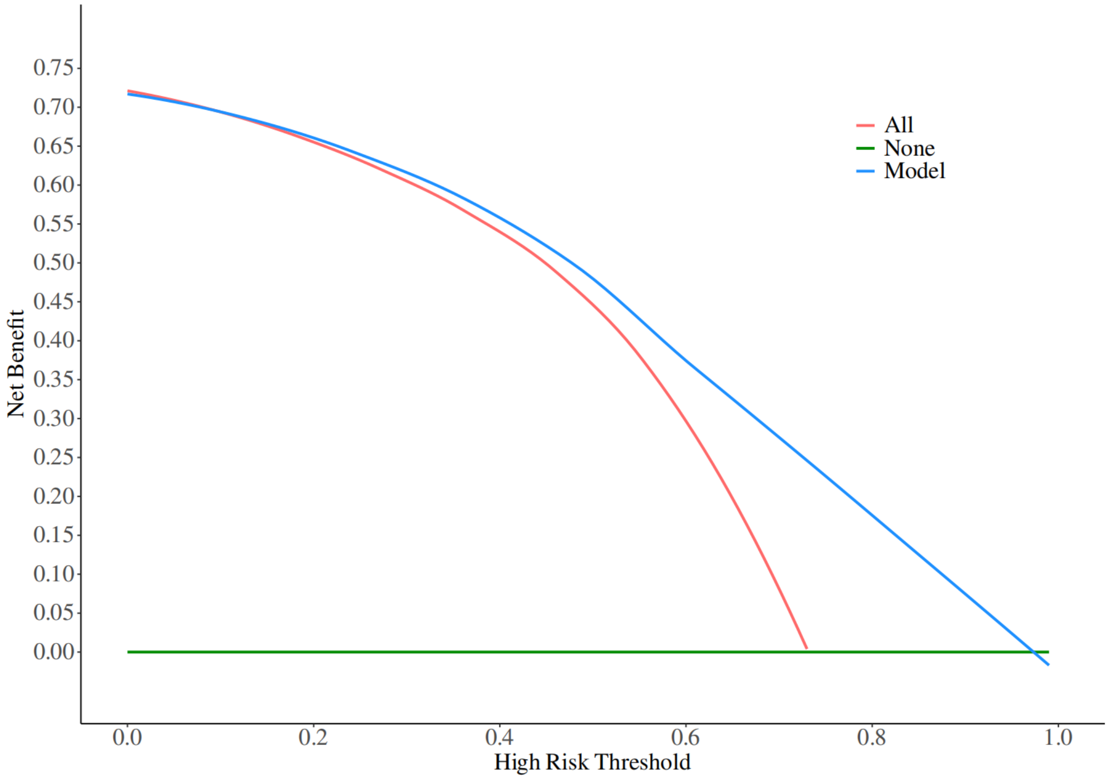

Figure legend: Decision curve analysis (DCA) for the test dataset. The blue line represents the net benefit of the prediction model across different threshold probabilities, compared with treating all patients (red line) or none (green line).

Supplementary Table S1. Frequency and distribution of embryonic chromosomal abnormalities

Chromosomal abnormality	N=163(%)
Single aneuploidy	118(72.4)
Autosomal trisomy	105(89.0)
Monosomy X	13(11.0)
Multiple aneuploidy	6(3.7)
Mosaicism of aneuploidy	5(3.1)
Triploidy	15(9.2)
Partial aneuploidy (large CNVs)	11(6.7)
Micro deletion/duplication	6(3.7)

Supplementary Table S2 Univariate regression analysis of included variables associated with Live birth

Variables	*P*	OR (95%CI)
Once abnormal chromosomal miscarriage, n(%)		
No		1.00 (Reference)
Yes	0.002	3.00 (1.50 ~ 6.01)
Once normal chromosomal miscarriage, n(%)		
No		1.00 (Reference)
Yes	0.008	0.40 (0.20 ~ 0.78)
Twice abnormal chromosomal miscarriage, n(%)		
No		1.00 (Reference)
Yes	0.147	4.61 (0.58 ~ 36.41)
Twice normal chromosomal miscarriage, n(%)		
No		1.00 (Reference)
Yes	0.042	0.17 (0.03 ~ 0.94)
No. of previous miscarriage	0.035	0.64 (0.43 ~ 0.97)
No. of parity		
0		1.00 (Reference)
1	0.877	1.06 (0.49 ~ 2.28)
≥2	0.785	1.40 (0.12 ~ 15.78)
No. of induced abortion		
0		1.00 (Reference)
1	0.121	1.86 (0.85 ~ 4.10)
≥2	0.406	1.86 (0.43 ~ 8.09)
No. of biochemistry pregnancy		
0		1.00 (Reference)
1	0.657	0.83 (0.37 ~ 1.86)
≥2	0.482	0.52 (0.08 ~ 3.21)
Maternal age (year)	0.272	1.04 (0.97 ~ 1.12)
Male age (year)	0.330	0.97 (0.90 ~ 1.04)
BMI (kg/m2)	0.584	1.04 (0.90 ~ 1.21)
AMH (ng/ml)	0.884	0.99 (0.83 ~ 1.18)
bFSH (IU/L)	0.678	1.05 (0.83 ~ 1.33)
bLH (IU/L)	0.768	0.97 (0.81 ~ 1.17)
bProlactin (IU/L)	0.540	0.99 (0.94 ~ 1.03)

Variables	*P*	OR (95%CI)
Fasting blood glucose (mmol/l)	0.518	1.34 (0.55 ~ 3.30)
TSH (mIU/L)	0.258	1.31 (0.82 ~ 2.09)
Mean gestational week (weeks)	0.776	0.97 (0.81 ~ 1.17)
TGAB (mU/L)	0.422	1.01 (0.99 ~ 1.03)
TPOAB (mU/L)	0.235	1.01 (0.99 ~ 1.04)
ANA (U/ml)	0.903	1.00 (0.97 ~ 1.04)
Months from the last POC CMA result to this pregnancy (months)	0.606	0.99 (0.95 ~ 1.03)

OR: Odds Ratio, CI: Confidence Interval
